# Large field of view, fast and low dose multimodal phase-contrast imaging at high x-ray energy

**DOI:** 10.1038/s41598-017-02412-w

**Published:** 2017-05-19

**Authors:** Alberto Astolfo, Marco Endrizzi, Fabio A. Vittoria, Paul C. Diemoz, Benjamin Price, Ian Haig, Alessandro Olivo

**Affiliations:** 1 Department of Medical Physics and Biomedical Engineering, University College London, Malet Place, Gower Street, London, United Kingdom; 2 X-Tek Systems-Nikon, Tring Business Centre, Icknield Way, Tring, Hertfordshire UK

## Abstract

X-ray phase contrast imaging (XPCI) is an innovative imaging technique which extends the contrast capabilities of ‘conventional’ absorption based x-ray systems. However, so far all XPCI implementations have suffered from one or more of the following limitations: low x-ray energies, small field of view (FOV) and long acquisition times. Those limitations relegated XPCI to a ‘research-only’ technique with an uncertain future in terms of large scale, high impact applications. We recently succeeded in designing, realizing and testing an XPCI system, which achieves significant steps toward simultaneously overcoming these limitations. Our system combines, for the first time, large FOV, high energy and fast scanning. Importantly, it is capable of providing high image quality at low x-ray doses, compatible with or even below those currently used in medical imaging. This extends the use of XPCI to areas which were unpractical or even inaccessible to previous XPCI solutions. We expect this will enable a long overdue translation into application fields such as security screening, industrial inspections and large FOV medical radiography – all with the inherent advantages of the XPCI multimodality.

## Introduction

X-ray imaging is one of the most widespread inspection/diagnostic techniques, mainly because of its simplicity and low cost. Its principles are straightforward: an x-ray source irradiates a sample, and an x-ray detector placed on the opposite side collects the transmitted photons. In general, x-rays allow the investigation of the internal features of opaque objects because of their reduced interaction with matter compared to electromagnetic radiation at longer wavelengths. This capability is widely exploited in application fields such as security screening, industrial inspections and medical diagnosis. While improvements have been introduced on the technical side, the basic principle of x-ray imaging has remained unchanged. In the last decades, however, x-ray phase contrast imaging (XPCI) has emerged as a new technique with the potential to improve the conventional x-ray imaging through a radically different principle. XPCI systems are sensitive to the x-ray phase-shift induced by the sample; this is described by δ in the complex refractive index n = 1 − δ + iβ, while the x-ray attenuation is related to β. Compared to attenuation, the phase contrast effect is significantly stronger for low Z materials (Z < 10, *e.g*. soft tissues), and it does not decrease as dramatically with increasing energy (E). XPCI is therefore considered an ideal candidate for applications involving low-Z materials, which generate a weak signal in conventional x-ray imaging. Moreover, XPCI could improve the applications where high x-ray dose could be an issue, since it allows to reduce it by increasing E while preserving high image contrast.

XPCI was first introduced by Bonse and Hart in 1965^1^, but became the subject of worldwide interest with the advent of ‘3^rd^ generation’ synchrotron radiation facilities^2–5^. Wilkins *et al*. demonstrated the possibility to perform XPCI using a conventional x-ray micro-focus source^6^, opening the way to a wider use in research. In the last decade, several XPCI techniques have been adapted and refined to be effective using conventional sources^7–9^. An important feature of most XPCI systems is their capability to simultaneously provide both attenuation and differential phase contrast (DPC) images, which makes conventional attenuation-based images still available when an XPCI setup is used. Moreover, a third contrast channel, often called dark-field (DF), can also be made available^10–14^. DF is x-ray refraction generated by details which cannot be resolved by the imaging system^10^, and it provides sub-pixel information on the microstructure of the object. Modern XPCI setups are effectively multimodal systems, capable of providing three contrast channels simultaneously. While the attenuation image provides the ‘classic’ x-ray information, DPC and DF images can be used to extend the range of applications of x-ray imaging. Indeed, XPCI proved enhanced performances on a wide range of applications including *ex-vivo* breast imaging^15–17^, pre-clinical lung disease investigation^18^ and detection of defects of composite materials^19^.

Despite significant research effort, XPCI setups still suffer from limitations which are preventing the technology to reach out beyond the research arena and be widely used in real-world applications. Typically, XPCI systems based on conventional x-ray sources require 2 or 3 micro fabricated optical elements (OEs) between the source and the detector (*i.e*. masks, gratings, *etc*.), which limit the field of view (FOV) to about 5 × 5 cm^2^. While larger FOVs can be obtained by stitching together several of those elements^20^, this increases the difficulties on aligning the system and, more importantly, it demands higher stability standards to keep the alignment reliable for long periods. A second key limitation of XPCI relates to the range of x-ray energies over which it can be made to work, which is typically skewed towards the low end of the spectrum. For example, so far breast imaging XPCI studies were performed with an unfiltered tungsten source at 40 kVp^17^, or with a molybdenum filtered molybdenum source at 40 kVp^15^. The pre-clinical lung studies were carried out at 35 kVp^18, 21^ or less^22^. In an example for non-destructive testing, XPCI was run using a tungsten source at 50 kVp^23^, one of the highest energies used to pursue a real application rather than for mere demonstration purposes. These low energies are normally chosen as a consequence of the poor performance of the OEs used in XPCI at high energies (*e.g*. the pre-sample and the detector masks in Edge-Illumination (EI) and the source and detector grating G0/G2 in Grating Interferometry (GI)). Those OEs rely on strong absorption materials (typically ‘as thick as possible’ gold layers) to improve the phase signal detection, minimizing the ‘Illumination-curve’ (IC) offset or maximizing the ‘visibility’ for EI and GI, respectively. At the moment, the relatively small pitch and aperture size of those absorbing OEs (a few microns for GI and tens of microns for EI, respectively) are limiting the thickness of the absorbing material (and therefore the accessible x-ray energy), because of the limitations in the maximum achievable aspect ratio, in particular for large FOV OEs. For this reason, XPCI applications are mostly limited to small objects imaged using small FOVs at low x-ray energies. As a consequence, several XPCI groups are trying to overcome those limitations. Tubes operated at 100 kVp were used first by Donath *et al*. (on a FOV of approximately 3 × 2 cm^2^)^24^, then by Ignatyev *et al*. with a 6 × 6 cm^2^ FOV^25^; Thüring *et al*. proposed an edge-on approach for the OE, which allowed the use of a tungsten tube at 160 kVp at the cost of an extremely reduced FOV in one direction (a single detector line) and long acquisition time (6 minutes per line)^26^. Sarapata *et al*. exploited a round OE of 10 cm in diameter to perform a Computed Tomography at 70 kVp^27^, and Horn *et al*. imaged a human knee at 90 kVp by stitching 17 × 18 small (15 mm × 35 mm) FOV images^28^. An alternative approach consists in removing the need for precise OEs and retrieving the phase information by using setups based on high magnification, as shown by Wang *et al*.^29^. They performed XPCI at 160 kVp using a steel wool as the OE, over a FOV of 5 × 4 cm^2^; however, this requires either a synchrotron or a microfocal source.

To the best of our knowledge, currently there are no conventional x-ray tube-based XPCI systems capable of simultaneously providing the following three desired features: 1- high x-ray energy; 2- large FOV and 3- fast acquisition time. While possibly some dedicated XPCI setups can be optimized to satisfy one or two of those characteristics, so far the simultaneous realization of all three seemed to be a technical impossibility. Applications fields such as baggage screening and industrial inspections could benefit enormously from an ‘ideal’ XPCI system, because they tend to require fast imaging of large and dense objects. Importantly, access to higher energies on a large FOV through fast scans would also open the way to the use of XPCI in *in vivo* medical applications on human patients.

In this paper we present a multimodal XPCI prototype based on EI that simultaneously satisfies all three key requirements outlined above through the development of an application-oriented XPCI system. This system can provide images of large objects (up to 20 × 50 cm^2^) up to 100 kVp^30^, and can be operated in ‘low-statistics’ mode to obtain XPC images of acceptable quality at 2.5 mm/s with a conventional x-ray source operated at only 2 mA. The prototype exploits the advantages of EI’s simple geometry (*i.e*. the x-ray masks’ large pitch and aperture size). The resulting relaxed aspect ratio allows realizing thicker structures, thus improving the quality of the OEs at high energy and relaxing the alignment requirements at the same time. The use of the asymmetric EI scanning solution^31^ eliminates the need of re-positioning any of the OEs during the acquisition to obtain the multimodal images; at the same time, it allows large FOVs. The system is flux efficient, because it allows for the use of a relatively large focal spot without additional source collimation *e.g*. source gratings. This guarantees good image quality also during a fast scan using a relatively low-power x-ray source. The system is also dose-efficient thanks to the main OE (pre-sample mask) being placed upstream of the sample. Both flux and dose efficiency are also enhanced by the use of low-absorbing graphite substrates.

## Results

**Figure 1:**
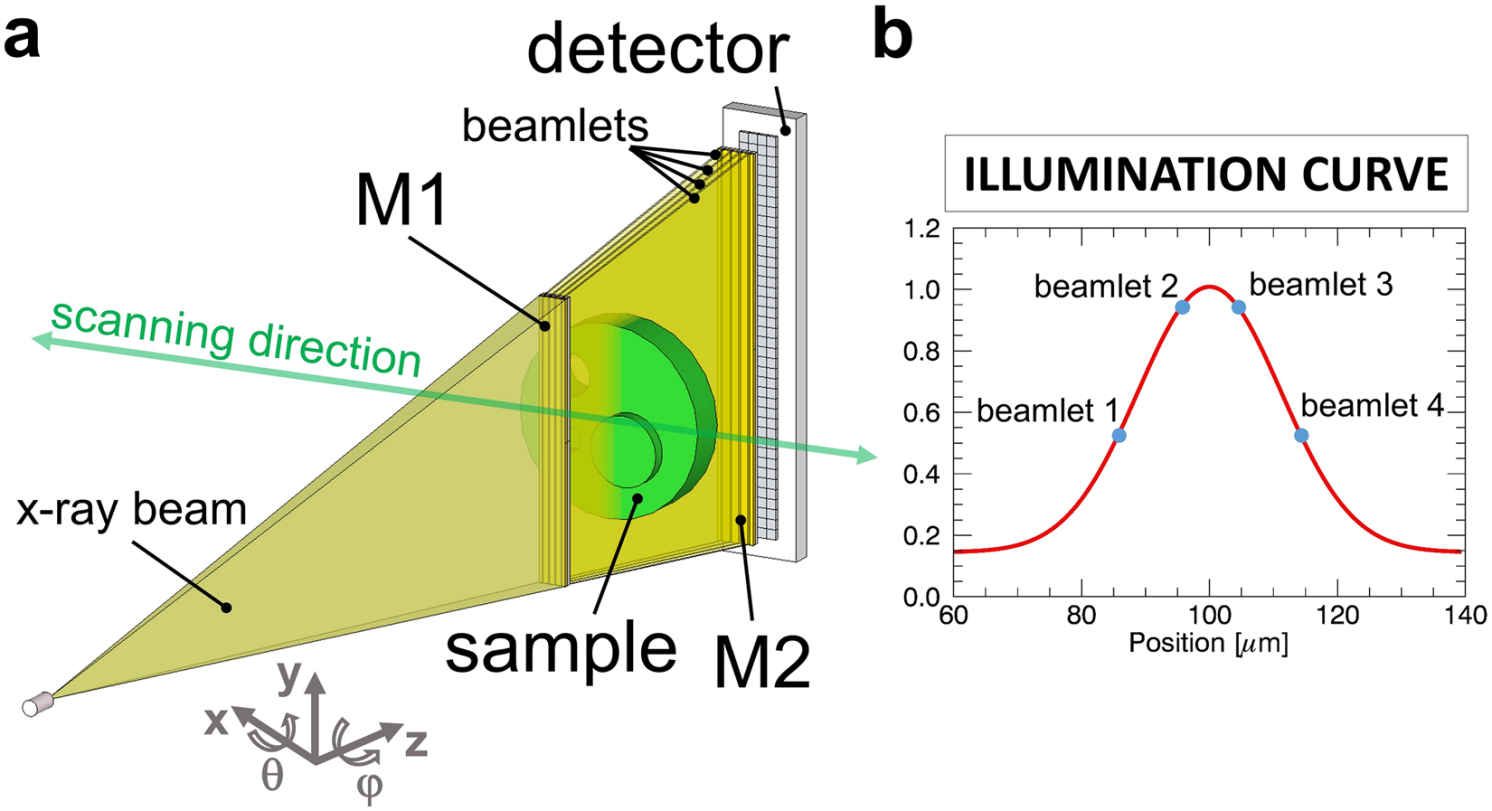
Schematic of the imaging system. Schematic view of the setup. The x-ray beam is shaped by the asymmetric pre-sample mask (M1) in several beamlets, realising different alignments with the apertures on the detector mask (M2) (**a**). During a sample scan each beamlet provides a separate full image from a specific point on the illumination curve (**b**).

A simplified scheme of the system is depicted in Fig. 1a. It requires two additional OEs if compared to conventional x-ray imaging setups. The x-ray beam is shaped by the pre-sample mask (M1) into small fan-shaped beamlets, which are aligned with the apertures of the detector mask (M2). The intensity curve in Fig. 1b (called Illumination Curve, IC) is obtained for each detector column by scanning M1 along the x-axis. In the asymmetric mask configuration^31^, each beamlet hits the detector mask on a different position, corresponding to a different value of the IC. In this way, only a lateral scan of the object is needed to obtain all the information necessary to retrieve absorption, DPC and DF images, and no movement of the OEs is required^31, 32^. The masks alignment in x, z, θ and φ is straightforward^33^, and was observed to be stable over several days of operations. Periodic checks (once every several acquisitions) of the M1 x-position are sufficient to guarantee artefact free images. This procedure is performed automatically when no samples are in the FOV.

**Figure 2:**
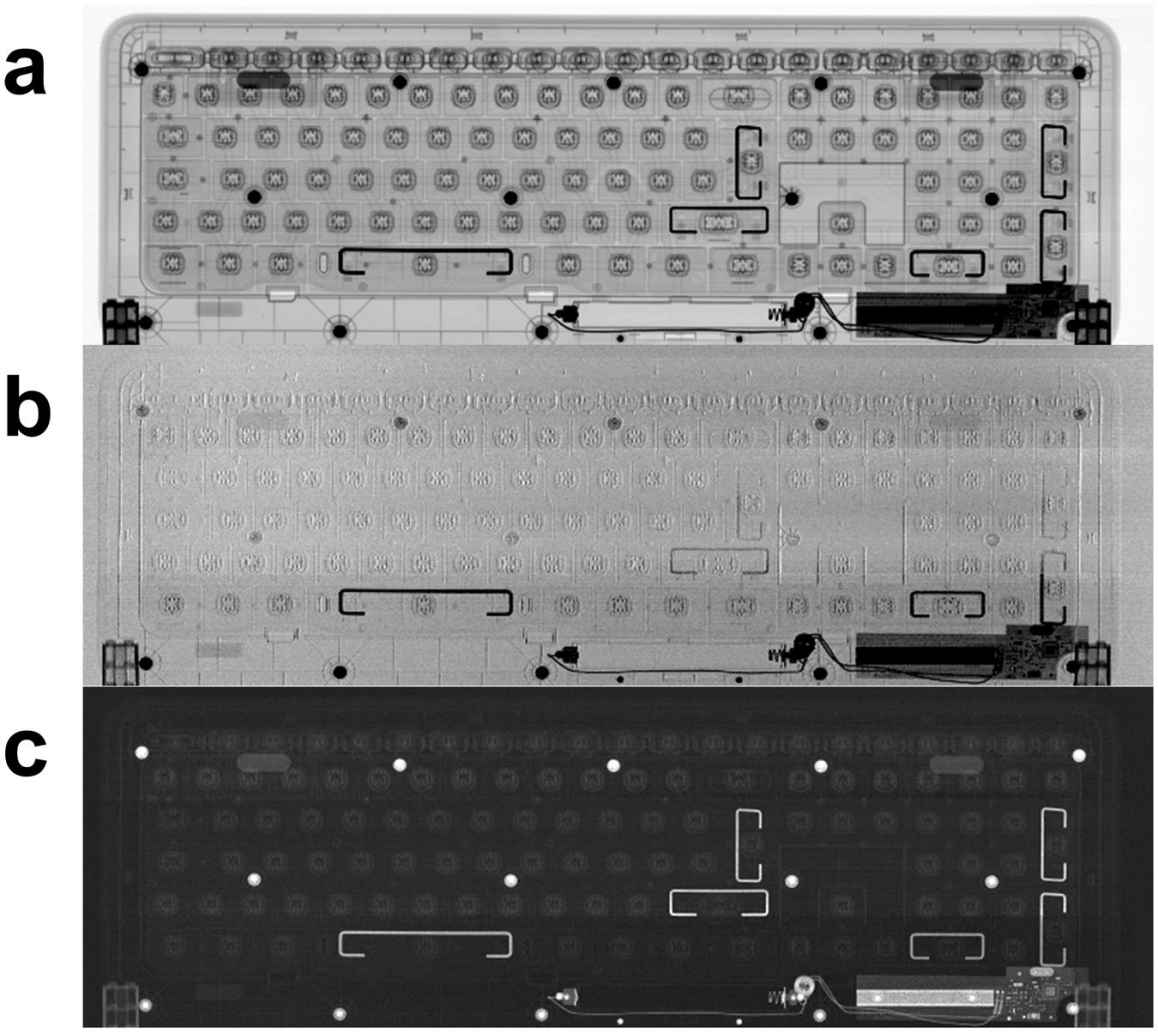
Example of a large field of view fast scan. The three retrieved images of a keyboard sample: **(a**) attenuation, (**b**) differential phase and (**c**) dark-field images (sample size 46 × 16 cm^2^; the sample was scanned along the longest (horizontal) direction).

**Figure 3:**
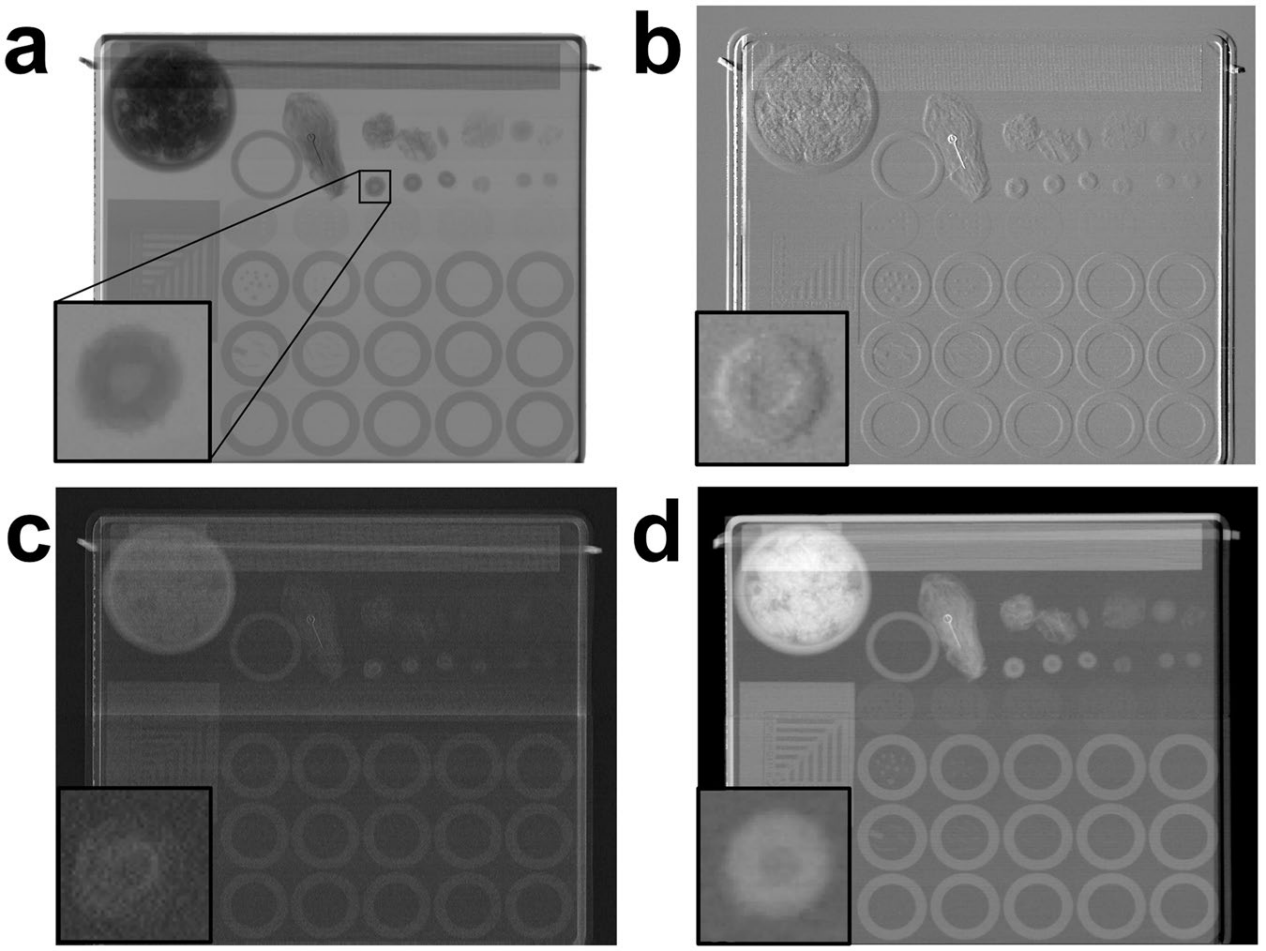
Example of a low x-ray dose scan. The three retrieved images of the mammographic Ackermann sample at low x-ray dose: (**a**) absorption, (**b**) differential phase, (**c**) dark-field images, and (**d**) phase map. The inset in the bottom left corner of each panel is a 5× zoom of the detail highlighted by the small square in panel (a) (sample size 10 × 11 cm^2^; the sample was scanned along the horizontal direction).

A first example of the system capabilities is shown in Fig. 2. Three complementary images of a full-sized computer keyboard were simultaneously obtained through a single 196 s scan at 80 kVp. The scan time can be significantly reduced further as explained in the discussion section, by using different equipment components which are however already available on the market. This shows easy adaptability to fast scanning of a variety of large objects in non-destructive testing and security applications. A second example aimed at demonstrating potential for low dose, full-field medical imaging (as an option for future development), is shown in Fig. 3. Here two example acquisitions of a standard Ackermann mammographic phantom^34^ are shown. The first three panels show a full, multimodal scan yielding absorption (a), DPC (b) and DF (c) images of the phantom obtained with an overall entrance dose of 2 mGy. Panel (d) shows a possible alternative use of the scanner in which multimodality is sacrificed in exchange for additional dose reduction through the use of a ‘single shot’ retrieval algorithm^35, 36^. In this case the entrance dose was only 0.15 mGy. These values should be compared with typical entrance dose levels of 10–12 mGy delivered in standard clinical mammography^37^. Note that an additional 2.4 cm Plexiglas layer was added to the phantom to reach an overall realistic thickness of 4.7 cm.

It should also be noted that the presented examples are aimed at demonstrating the capabilities of the system; specific applications of those capability will then have to be investigated and optimized on a case-by-case basis.

## Discussion

The developed system allows for the first time performing XPCI on large FOVs using a high-energy beam in a short acquisition time. The optimized use of EI was key to solve most of the technical limitations. One of the key reason is EI’s inherent technical simplicity compared to other approaches^8^. The main factor contributing to this simplicity is the relatively large pitch (70 ÷ 100 µm; matching the detector pixels pitch) and aperture size (10 ÷ 30 µm) in the OEs. For the same aspect ratio, this allows increasing their gold thickness, thus improving the technique’s effectiveness at higher x-ray energies. It also makes the system much more robust against instabilities and easier to align. Those are important features for applications such as security screening or industrial quality controls, where the time spent to realign the system translates into a loss in efficiency/productivity since throughput is key. The large feature size in the OEs also made it easy to align three smaller masks in order to obtain a system with larger FOV, with negligible “blind” areas between the individual elements. Minimal artefacts due to some residual misalignment among the individual mask elements are noticeable though a careful investigation. A second crucial advantage came from the asymmetric EI implementation. Typically, XPCI requires repositioning and/or scanning one of the OEs and acquiring several frames to obtain the data necessary for retrieving the DPC and DF information. In asymmetric EI, only the sample scan is required while everything else remains stationary. This allows speeding up the acquisition and simplifying the entire process. EI is also an efficient technique in terms of dose delivery. The absorbing first mask protects the sample from the unused parts of the direct beam, meaning that the sample is irradiated only by the fan-shaped beamlets. This makes the system well suited for dose-critical applications in medical screening and diagnostic procedures.

As an example of the system capabilities, a large common object was scanned at high energy in approximately 3 minutes (Fig. 2). It should be noted that the tube current was only 2 mA, leaving ample margin for additional increase in scanning speed. Moreover, a total of 128 detector columns were used, and speed can also be increased by using a larger number of detector columns thus matching full use of the cone beam. This system therefore makes DPC and DF available at high energy and over large FOVs in short exposure times, allowing a straightforward translation of XPCI in fields such as security screening, industrial inspections, medical screening (chest, dental, breast, possibly even full-body radiography), which were all not accessible with XPCI before. Our results also open the possibility to explore the benefits of XPCI mammography at higher energy, which can lead to very significant dose reductions as recently demonstrated by a pilot study with synchrotron radiation^36^. The low-dose x-ray images shown in Fig. 3 provide preliminary evidence that it may be possible to add to the information available with conventional mammography (*i.e*. attenuation), by making DPC and DF images available while still operating at a reduced dose compared to current clinical standards. The latter image has been proved beneficial for enhanced diagnosis^16, 38^. Additional very significant dose reductions can be obtained using a single-shot phase-retrieval algorithm^35^, which provides a phase map of the object (Fig. 3d), under sample homogeneity assumptions which are well met by breast tissue.

It should also be noted that further improvements of the setup are still possible. For example, the x-ray tube was operated at 160 W, about an order of magnitude below standard practice in XPCI where tube powers above 1 kW are normally used^16, 26, 27^, including with comparable focal spots^15, 39^. Such a source could be used to speed up the acquisition, increase image quality or for a combination of both. The focal spot could also be extended along one direction without significant negative effects in the (orthogonal) direction of phase sensitivity, and it has recently been demonstrated that the use of multiple sources (obtained *e.g*. by sectioning a larger source, similarly to what done in grating interferometry when a “source grating” is used) is also possible^40^. Another gain in photon flux can be achieved by reducing the distance between the source and the detector (currently at 2 m), and higher statistics can be achieved by increasing the number of detector columns used to create the final images (currently 128 or 1.28 cm, which does not cover the whole available beam cross-section). Energy can be further increased if higher aspect ratio masks (>10:1) are used, which can for example be obtained by adapting and optimizing metal-assisted chemical etching silicon techniques already in use for the small OEs in GI, where exceptionally high aspect ratios of gold structures such as 16:1^41^ or even 40:1^42^ have been produced.

In summary, we have designed, built and tested the first system capable of high energy, large FOV and fast XPCI. So far XPCI has been used mainly at low energies, small FOV and required slow acquisitions, which restricted its application remit to research projects. The development presented in this paper allows XPCI applications which were not possible before. Importantly the new system can be straightforwardly adapted for use in a real-world application through minimum engineering investment, thus realising a long-awaited commercial translation of XPCI.

It should be noted here that much faster acquisitions are routinely achieved at synchrotron radiation facilities^43, 44^, however here the focus is on translational aspects, which make the use of conventional sources mandatory.

## Methods

### Asymmetric Edge Illumination

In ‘classic’ EI the x-ray beam is shaped in thin fan beamlets by a highly absorbing gold pre-sample mask (M1 in Fig. 1a) with symmetric apertures. Each beamlet is aligned with a second (detector) mask (M2 in Fig. 1a) with matching apertures; every beamlet aperture pair combination corresponds to a detector column. A lateral scan of M1 provides a bell-shaped IC (Fig. 1b) for each pixel. This has a maximum when corresponding apertures are aligned and two close-to-zero minima for full misalignment at the left and right hand sides of the maximum^8^. An object placed between M1 and M2 has three effects on the IC: 1- absorption, changing the area below the IC; 2- DPC, changing the position of the IC maximum; 3- DF, changing the width of the IC. The retrieval of absorption, DPC and DF requires at least three images acquired at different positions of the IC^14^. In asymmetric EI, subgroups of apertures in M1 are slightly laterally shifted from their symmetric positions^31^. In this way, each detector column provides a full object image on a specific position of the IC through an object scan, without the need to move any of the OEs. The shifted aperture subgroups were designed in such a way that every sub-group will acquire images at one of the points highlighted with circles in Fig. 1b. Images from columns belonging to the same sub-group are then summed together to increase the statistics, and fed to the retrieval algorithm, ultimately providing attenuation, DPC and DF images.

### Experimental system

The system was designed making use of experimentally validated Monte Carlo simulations with the goal of optimizing the output for absorption, DPC and DF^45–47^. It was built inside a stand-alone lead-shielded cabinet at the Nikon Metrology UK’s factory in Tring, Hertfordshire, UK. It is based on a Tungsten X-Tek 160 tube with a focal spot tuned to be approximately 80 µm. M1 and M2 masks are placed at 1.5 m and 1.95 m from the source, respectively. The two masks are realized by electroplating 200 µm of gold on 500 µm thick graphite substrates. Both large masks are a combination of 3 smaller masks carefully aligned and fixed on a steel frame. The resulting sizes are 1.2 × 15 cm^2^ for M1, and 1.5 × 20 cm^2^ for M2. The detector is a dual-energy single photon counter Cd-Te CMOS (XCounter XC-FLITE FX2) with 2048 × 128 square pixels 100 µm in side, placed 2 m from the x-ray source. The two masks frames are mounted on two separate 4-axis stepper motor turrets for alignment. Apertures are 21.4 µm and 28 µm for the smaller and the larger mask, respectively, with their projected pitches matching the detector pixels (*i.e*. actual pitches are 75 µm and 97.5 µm respectively). Apertures in M1 are arranged in asymmetric groups of four, in order to generate 4 separate images on the IC in a single object scan^31^ (see Fig. 1b). The shifts are: -s, 0, s and 2 × s with s = 10 µm. The system’s spatial resolution in the vertical direction is determined by the detector’s effective pixel (75 µm), or any of its multiples if used in rebinned mode. The resolution in the scanning direction can be tuned by varying the scanning speed and the acquisition frame rate, with a minimum equal to the aperture sizes in M1 *i.e*. 21.4 µm^48^.

### Samples and acquisition parameters

The keyboard in Fig. 2 is a standard wireless keyboard (Microsoft Wireless Keyboard 800; 46 × 16 cm^2^). The source was set at 80 kVp and 2 mA without filters. The detector acquisition rate was 32 Hz and the acquisition speed was 2.5 mm/s, leading to a total scan time of 198 s. The effective acquisition resolution is 75 µm × 75 µm. The relatively high resolution in the acquisition is needed to detect the edge-enhancement peaks. Images can then be resized after data reconstruction. For example, images in Fig. 2 are rebinned to 600 µm × 600 µm. To downsize the differential phase image, a selective sampling to preserve minima and maxima was performed as opposed to rebinning; note however that the reported acquisition time corresponds to the original acquisition with 75 µm step. The second sample is the Ackermann mammographic phantom (RMI 160; Gammex, Middleton, WI, USA) with size 10 × 11 × 2.3 cm^3^, which contains a series of simulated lesions^34^. It has been acquired together with a 2.4 cm PMMA slab to simulate a realistic compressed breast thickness of 4.7 cm. For this application, we tested the system using two different settings. In the first case (Fig. 3a,b and c), the tube was set at 80 kVp and 2 mA, and the beam was filtered with 1 mm of Al. The effective pixel in the acquisition was 75 µm × 75 µm. The x-ray entrance dose was 2 mGy, as measured using a calibrated and certified dosimeter (UNIDOS E PTW – Universal dosimeter equipped with a soft x-ray chamber type 23344). The second acquisition (Fig. 3d) used an x-ray beam at 56 kVp, a current of 2 mA and 1 mm of Al filtration. This time the effective pixel in the acquisition was 37.5 µm × 75 µm. Both acquisitions had a total scan time of approximately 12 minutes. It should be noted that the increased scan time is due to the desire to reach clinically significant doses with the low dose rate provided by the used source. This is not only due to its limited power (see comments above), but also to the relatively high x-ray energy employed in this case compared to a standard (Molybdenum) mammographic spectrum. We simulated an acquisition at a lower entrance dose using only one of the 4 images from the asymmetric groups of apertures. In this case the entrance dose was 0.15 mGy (Fig. 3d). Conversely, the samples in Figs 2 and 3a,b and c were acquired using images from all 4 symmetric positions on the IC (two on one side and two on the other as shown in Fig. 1b). This choice of points simplifies the reconstruction formulas while providing sufficiently high image quality.

### Data reconstruction

The raw data from each column of the detector are flat-fielded using images recorded without the sample before and after each sample scan. Images from each of the 4 groups of apertures (corresponding to the different asymmetric values: -s, 0, s and 2 × s, see above) are then shifted and added together to increase statistics (with a total of 32 images per asymmetric group). The used retrieval algorithm is an adaptation to the 4-points case of the method first proposed by Rigon *et al*. for analyser-based imaging^49, 50^ and later extended to GI^51^. Following the same formalism, the 4 normalized images obtained (I_j_) can be expressed as a second order Taylor expansion for the IC, $R(\,\,\bar{\varphi }+{\rm{\Delta }}{\varphi }_{R}+{\rm{\Delta }}{\varphi }_{S})$ around $\bar{\varphi }$, where Δ*ϕ*_*R*_ is the refraction angle and Δ*ϕ*_*s*_ is the stochastic scattering angle:

1 $${I}_{j}={I}_{R}[{R}_{j}+{\dot{R}}_{j}{\rm{\Delta }}{\varphi }_{R}+\frac{1}{2}{\ddot{R}}_{j}{\rm{\Delta }}{{\varphi }_{R}}^{2}+\frac{1}{2}{\ddot{R}}_{j}{\sigma }_{{\rm{\Delta }}{\varphi }_{S}}^{2}],\,j\in [1,4]$$

Here, *R*_*j*_, ${\dot{R}}_{j}$ and ${\ddot{R}}_{j}$ are the value, the first derivative and the second derivative of the IC calculated at the point $\overline{{\varphi }_{j}}$, respectively. *I*_*R*_, Δ*ϕ*_*R*_ and ${\sigma }_{{\rm{\Delta }}{\varphi }_{S}}^{2}$ are the pixel-wise absorption, DPC and DF signals induced by the imaged object, respectively^49^. The 4-equations system can be solved to obtain:

2 $${I}_{R}=\frac{1}{2}\frac{{I}_{1}+{I}_{2}+{I}_{3}+{I}_{4}}{{R}_{1}+{R}_{2}}$$

3 $${\rm{\Delta }}{\varphi }_{R}=\frac{({I}_{1}+{I}_{2})-({I}_{3}+{I}_{4})}{{I}_{1}+{I}_{2}+{I}_{3}+{I}_{4}}\frac{{R}_{1}+{R}_{2}}{{\dot{R}}_{1}+{\dot{R}}_{2}}$$

4 $${\sigma }_{{\rm{\Delta }}{\varphi }_{S}}^{2}=2\frac{{R}_{1}({I}_{2}+{I}_{3})-{R}_{2}({I}_{1}+{I}_{4})}{{\ddot{R}}_{2}({I}_{1}+{I}_{4})}-{\rm{\Delta }}{\varphi }_{R}^{2}$$

In analogy with the cited work by Rigon *et al*., the following assumptions were made: - small refraction and DF angles, symmetric images on the IC with images 1 and 4 at ±50% of the IC. We note that those assumptions are satisfied in most practical cases, especially at high x-ray energies like those employed in this study, since they lead to smaller refraction and DF angles. However, alternative approaches such as IC fitting^14, 47^ can be used in situations where those assumptions are not fulfilled. The ultra-low x-ray dose image in Fig. 3d was processed accordingly to the algorithm presented by Diemoz *et al*.^35, 36^ starting from a single image on the IC (beamlet 1 in Fig. 1b). The image represents the phase map of the object.

### Data Availability

The datasets generated during and/or analysed during the current study are available from the corresponding author on reasonable request.

## References

[1] Bonse U, Hart M (1965). AN X-RAY INTERFEROMETER. Applied Physics Letters.

[2] Momose A (1995). Phase-Contrast Radiographs of Nonstained Rat Cerebellar Specimen. Medical Physics.

[3] Momose A, Takeda T, Itai Y, Hirako K (1996). Phase-contrast X-ray computed tomography for observing biological soft tissues. Nature Medicine.

[4] Chapman D (1997). Diffraction enhanced x-ray imaging. Physics in Medicine and Biology.

[5] Cloetens P (1997). Observation of microstructure and damage in materials by phase sensitive radiography and tomography. Journal of Applied Physics.

[6] Wilkins SW, Gureyev TE, Gao D, Pogany A, Stevenson AW (1996). Phase-contrast imaging using polychromatic hard X-rays. Nature.

[7] Pfeiffer F, Weitkamp T, Bunk O, David C (2006). Phase retrieval and differential phase-contrast imaging with low-brilliance X-ray sources. Nature Physics.

[8] Olivo, A. & Speller, R. A coded-aperture technique allowing x-ray phase contrast imaging with conventional sources. *Applied Physics Letters* **91**, doi:10.1063/1.2772193 (2007).

[9] Parham C, Zhong Z, Connor DM, Chapman LD, Pisano ED (2009). Design and Implementation of a Compact Low-Dose Diffraction Enhanced Medical Imaging System. Academic Radiology.

[10] Rigon L (2003). A new DEI algorithm capable of investigating sub-pixel structures. Journal of Physics D: Applied Physics.

[11] Pagot E (2003). A method to extract quantitative information in analyzer-based x-ray phase contrast imaging. Applied Physics Letters.

[12] Wernick MN (2003). Multiple-image radiography. Physics in Medicine and Biology.

[13] Pfeiffer F (2008). Hard-X-ray dark-field imaging using a grating interferometer. Nature Materials.

[14] Endrizzi, M. & Olivo, A. Absorption, refraction and scattering retrieval with an edge-illumination-based imaging setup. *Journal of Physics D: Applied Physics* **47**, doi:10.1088/0022-3727/47/50/505102 (2014).

[15] Olivo, A. *et al*. Low-dose phase contrast mammography with conventional x-ray sources. *Medical Physics* **40**, doi:10.1118/1.4817480 (2013). 10.1118/1.4817480 24007133

[16] Wang, Z. *et al*. Non-invasive classification of microcalcifications with phase-contrast X-ray mammography. *Nature Communications* **5**, doi:10.1038/ncomms4797 (2014). 10.1038/ncomms4797 24827387

[17] Stampanoni M (2011). The first analysis and clinical evaluation of native breast tissue using differential phase-contrast mammography. Investigative Radiology.

[18] Velroyen A (2015). Grating-based X-ray Dark-field Computed Tomography of Living Mice. EBioMedicine.

[19] Endrizzi M, Murat BIS, Fromme P, Olivo A (2015). Edge-illumination X-ray dark-field imaging for visualising defects in composite structures. Composite Structures.

[20] Meiser J (2016). Increasing the field of view in grating based X-ray phase contrast imaging using stitched gratings. Journal of X-Ray Science and Technology.

[21] Hellbach K (2016). Facilitated Diagnosis of Pneumothoraces in Newborn Mice Using X-ray Dark-Field Radiography. Investigative Radiology.

[22] Bech, M. *et al*. *In-vivo* dark-field and phase-contrast x-ray imaging. *Scientific Reports* **3**, doi:10.1038/srep03209 (2013). 10.1038/srep03209 PMC3826096 24220606

[23] Uehara, M., Yashiro, W. & Momose, A. Effectiveness of X-ray grating interferometry for non-destructive inspection of packaged devices. *Journal of Applied Physics* **114**, doi:10.1063/1.4823982 (2013).

[24] Donath, T. *et al*. Phase-contrast imaging and tomography at 60 keV using a conventional x-ray tube source. *Review of Scientific Instruments* **80**, doi:10.1063/1.3127712 (2009). 10.1063/1.3127712 19485510

[25] Ignatyev, K., Munro, P. R. T., Chana, D., Speller, R. D. & Olivo, A. Coded apertures allow high-energy x-ray phase contrast imaging with laboratory sources. *Journal of Applied Physics* **110**, doi:10.1063/1.3605514 (2011).

[26] Thüring, T., Abis, M., Wang, Z., David, C. & Stampanoni, M. X-ray phase-contrast imaging at 100 keV on a conventional source. *Scientific Reports* **4**, doi:10.1038/srep05198 (2014). 10.1038/srep05198 PMC4047533 24903579

[27] Sarapata A (2015). Quantitative imaging using high-energy X-ray phase-contrast CT with a 70 kVp polychromatic X-ray spectrum. Optics Express.

[28] Horn, F. *et al*. In Progress in Biomedical Optics and Imaging - Proceedings of SPIE.

[29] Wang, H., Kashyap, Y., Cai, B. & Sawhney, K. High energy X-ray phase and dark-field imaging using a random absorption mask. *Scientific Reports* **6**, doi:10.1038/srep30581 (2016). 10.1038/srep30581 PMC4964655 27466217

[30] Astolfo, A., Endrizzi, M., Price, B., Haig, I. & Olivo, A. In *Proceedings of SPIE - The International Society for Optical Engineering*., Vol. 9995, doi:10.1117/12.2239556 (2016)

[31] Endrizzi, M., Astolfo, A., Vittoria, F. A., Millard, T. P. & Olivo, A. Asymmetric masks for laboratory-based X-ray phase-contrast imaging with edge illumination. *Scientific Reports* **6**, doi:10.1038/srep25466 (2016). 10.1038/srep25466 PMC4857105 27145924

[32] Kottler, C., Pfeiffer, F., Bunk, O., Grünzweig, C. & David, C. Grating interferometer based scanning setup for hard x-ray phase contrast imaging. *Review of Scientific Instruments* **78**, doi:10.1063/1.2723064 (2007). 10.1063/1.2723064 17477673

[33] Millard, T. P. *et al*. Method for automatization of the alignment of a laboratory based x-ray phase contrast edge illumination system. *Review of Scientific Instruments* **84**, doi:10.1063/1.4816827 (2013). 10.1063/1.4816827 PMC7116146 24007068

[34] Arfelli F (2000). Mammography with synchrotron radiation: Phase-detection techniques. Radiology.

[35] Diemoz PC (2015). Single-image phase retrieval using an edge illumination X-ray phase-contrast imaging setup. Journal of Synchrotron Radiation.

[36] Diemoz PC (2016). A method for high-energy, low-dose mammography using edge illumination x-ray phase-contrast imaging. Physics in Medicine and Biology.

[37] Gennaro G, Baldelli P, Taibi A, Di Maggio C, Gambaccini M (2004). Patient dose in full-field digital mammography: An italian survey. European Radiology.

[38] Scherer, K. *et al*. Improved Diagnostics by Assessing the Micromorphology of Breast Calcifications via X-Ray Dark-Field Radiography. *Scientific Reports* **6**, doi:10.1038/srep36991 (2016). 10.1038/srep36991 PMC5107908 27841341

[39] Marenzana M (2012). Visualization of small lesions in rat cartilage by means of laboratory-based x-ray phase contrast imaging. Physics in Medicine and Biology.

[40] Basta, D., Endrizzi, M., Vittoria, F. A., Astolfo, A. & Olivo, A. Compact and cost effective lab-based edge-illumination x-ray phase contrast imaging with a structured focal spot. *Applied Physics Letters* **108**, doi:10.1063/1.4953459 (2016).

[41] Li L (2016). A facile and low-cost route to high-aspect-ratio microstructures on silicon: Via a judicious combination of flow-enabled self-assembly and metal-assisted chemical etching. Journal of Materials Chemistry C.

[42] Romano L, Kagias M, Jefimovs K, Stampanoni M (2016). Self-assembly nanostructured gold for high aspect ratio silicon microstructures by metal assisted chemical etching. RSC Advances.

[43] Uesugi K, Sera T, Yagi N (2006). Fast tomography using quasi-monochromatic undulator radiation. Journal of Synchrotron Radiation.

[44] Mokso, R. *et al*. Four-dimensional *in vivo* X-ray microscopy with projection-guided gating. *Scientific Reports* **5**, doi:10.1038/srep08727 (2015). 10.1038/srep08727 PMC4356984 25762080

[45] Bergbäck Knudsen E (2013). McXtrace: A Monte Carlo software package for simulating X-ray optics, beamlines and experiments. Journal of Applied Crystallography.

[46] Millard, T. P., Endrizzi, M., Diemoz, P. C., Hagen, C. K. & Olivo, A. Monte Carlo model of a polychromatic laboratory based edge illumination x-ray phase contrast system. *Review of Scientific Instruments* **85**, doi:10.1063/1.4873328 (2014). 10.1063/1.4873328 24880377

[47] Astolfo A (2016). A first investigation of accuracy, precision and sensitivity of phase-based x-ray dark-field imaging. Journal of Physics D: Applied Physics.

[48] Diemoz PC, Vittoria FA, Olivo A (2014). Spatial resolution of edge illumination X-ray phase-contrast imaging. Optics Express.

[49] Rigon L, Arfelli F, Menk RH (2007). Generalized diffraction enhanced imaging to retrieve absorption, refraction and scattering effects. Journal of Physics D: Applied Physics.

[50] Rigon, L., Arfelli, F. & Menk, R. H. Three-image diffraction enhanced imaging algorithm to extract absorption, refraction, and ultrasmall-angle scattering. *Applied Physics Letters* **90**, doi:10.1063/1.2713147 (2007).

[51] Pelliccia D (2013). A three-image algorithm for hard x-ray grating interferometry. Optics Express.

